# Use of an Acellular Assay to Study Interactions between Actinides and Biological or Synthetic Ligands

**DOI:** 10.3390/biom12111553

**Published:** 2022-10-24

**Authors:** Anne Van der Meeren, Catherine Berthomieu, Agnès Moureau, Martine Defrance, Nina M. Griffiths

**Affiliations:** 1 Laboratory of Radio Toxicology, Commissariat à l’énergie atomique et aux energies alternatives (CEA), Paris-Saclay University, 91297 Arpajon, France; 2 Protein-Metal Interactions Laboratory, Commissariat à l’énergie Atomique et aux Energies Alternatives (CEA), Aix Marseille University, Centre National de Recherche Scientifique (CNRS), 13108 Saint Paul-Lez-Durance, France

**Keywords:** actinides, bioavailability, bioligands, protein interaction, chelation

## Abstract

Speciation of actinides, and more particularly bioligand-binding ability, influences in vivo behavior. Understanding these interactions is essential for estimation of radiological dose and improvement of decorporation strategies for accidentally contaminated victims. Because the handling of actinides imposes overwhelming difficulties, in vitro assays carried out in physiological conditions are lacking and data regarding such interactions are scarce. In this study, we used a bi-compartmental and dynamic assay, providing physiological conditions (presence of inorganic ions, pH, temperature) to explore interactions between the actinides plutonium (Pu) and americium (Am) and endogenous (proteins transferrin and ferritin) or exogenous ligands (the chelating agent diethylenetriaminpentaacetic acid, DTPA). In this assay, an agarose gel represents the retention compartment of actinides and a dynamic fluid phase, the transfer compartment. The proportion of actinides transferred from static to dynamic phase reflects interactions between Pu/Am and various ligands. The results show differences in the formation of actinide-protein or actinide-DTPA complexes in physiologically relevant media depending on which ligand is present and where. We observed differential behavior for Pu and Am similar to in vivo studies. Thus, our assay may be used to determine the ability of various actinides to interact with specific proteins or with drug candidates for decorporation in complex physiologically relevant environments.

## 1. Introduction

Internal contamination with actinides (An) is a potential hazard concerning both workers in the nuclear industry and larger populations in case of nuclear reactor accidents or intentional release as a consequence of a malevolent act. Within the actinide series, plutonium (Pu) and americium (Am) are of specific concern since they are produced in large quantities in nuclear reactors, have long lived isotopes, and are high-energy alpha particle emitters. Retention of Pu or Am in the body will result in local irradiation of the tissue and subsequent pathology. Actinides can enter the body through different routes, mainly by inhalation or through wounds, and under various physicochemical forms. Physical properties highly influence the way An distribute in the different organs after their penetration in the body. The proportion of inhaled An transferred to blood depends on the speciation and the solubility of the compound, namely its bioavailability [1]. The more soluble compounds have the shortest retention time at the contamination site and the fastest transfer to blood [2]. The initial status of An may be modified according to the local environment encountered at the site of contamination. Then, after transfer into the circulation, the behavior of the An will strongly depend on its affinity for specific biological ligands which influences the sequestration in specific intra- or extra-cellular compartments [3]. The interactions with blood components depend on the chemical nature of the An, and in turn, influence the body distribution and determine the systemic target tissues, i.e., predominantly liver and bones for Pu and Am. These differences in speciation and in endogenous bioligand binding, are important to be understood, as they will also modify the bioaccessibility to exogenous chelating agents. This is illustrated by the differential efficacy of the currently approved chelating drug for actinide decorporation, i.e., diethylenetriaminpentaacetic acid (DTPA) according to the route of An Intake and its physicochemical form [4]. Despite the higher affinity of DTPA towards Pu (logK 29–36) as compared to Am (logK 23–26) (for reviews, see [1,5]), the higher efficacy of DTPA for the decorporation of Am as compared to Pu is generally attributed to the higher in vivo bioavailability of Am [6,7]. In line with this hypothesis, Paquet and colleagues demonstrated a better decorporation of Am as compared to Pu using the octadentate ligand 3,4,3-LI(1,2-HOPO) following wound contamination with MOX nuclear fuel [8]. Actinides do not usually remain as simple ions at physiological pH and in the presence of biological ligands [9]. Complexes may be formed with various endogenous ligands which can be inorganic ions (hydroxyl, citrate, carbonate, phosphate, etc.) or proteins with affinity for the element. These factors are particularly important for Pu, which displays a very complex redox chemistry and a strong tendency to hydrolyze at physiological pH. Although they both belong to the actinide series, Pu and Am exhibit different chemical properties, such as their oxidation states under physiological conditions (Pu (IV) and Am (III)) and slightly different coordination properties [9]. These characteristics determine their interactions with ligands and consequently their biological behavior and distribution. Retention times in various compartments in the body vary significantly between Pu and Am [2] such as the much faster removal of Am from blood [10,11].

It has been recognized for decades that Pu forms very strong complexes with transferrin (Tf) particularly in the plasma, where Tf is the main carrier protein for Pu (90%), whereas only 30% of Am is found under Am-Tf complexes [12,13]. The lower proportion of Am-Tf complexes in the blood could be explained by their lower stability as compared to Pu-Tf ones [14]. Of interest is a recent study showing that Tf and ferritin were differentially involved in the cellular uptake of Pu and Am in an in vitro model of lung epithelial cells [15]. In this study, the authors demonstrated that complexation with Tf and ferritin appears to block cellular uptake by lung epithelial cells, which was not the case for Am. An additional difference between Pu and Am concerns the distribution in the systemic organs of retention (skeleton:liver ratio) and its time-related evolution [14,16]. In addition, Pu is preferentially localized on endosteal bone surfaces, while Am is deposited more uniformly on both endosteal and periosteal surfaces [17,18]. Furthermore, transfer to the bone marrow was observed to be higher for Pu than for Am, which could possibly lead to a different incidence or type of damage [19]. Ferritin represents another protein with strong affinity towards An particularly in the liver [20]. In addition, Pu(IV) transfer from transferrin to ferritin has been described in vitro [21].

Characterization of actinide-bioligand interactions is of great interest to understand how An are distributed in the body, how they are transported, and how they penetrate organs or cells. A few publications report studies of Pu/ Am-protein interactions [12,22,23,24,25,26,27,28,29,30,31] but most of the stability constants of the Pu/Am-protein complexes are not known except for Pu-Tf [24,25] and Pu-fetuin [28]. In addition, An-protein complex formation is often estimated in purely chemical and non-physiological conditions. The lack of data and the scarcity of studies may result from the difficulty carrying out An studies in physiologically relevant environments as well as the lack of experimental methods that cause minimal disturbance to the natural speciation of the system (pH, salt concentration, temperature). Indeed, complex chemistry, high reactivity, and highly radioactive properties of these elements render their study particularly challenging, and may explain the use of surrogates such as thorium (Th) or cerium (Ce) for Pu or samarium (Sm) for Am. However, these surrogates appear to be not always representative as previously reported [32,33].

To improve the characterization of An-bioligand interactions in more physiologically relevant environments, we recently developed a biphasic and dynamic in vitro acellular assay [34,35]. This assay mimics the transfer of bioavailable An species from a retention compartment (agarose gel) to a dynamic fluid phase (transfer compartment) under stirring at 37 °C and in a 5% CO_2_ atmosphere. The whole volume of the dynamic phase is removed at 2, 24, and 48 h and replaced by fluid of the same composition. Relevant physiological conditions are obtained by introducing various components into both the static and dynamic phases. Our previous work suggested that this assay could reflect interactions occurring in biological surroundings when the static phases are made or incubated in the presence of potential ligands, either endogenous or exogenous. Thus, we were able to demonstrate, in accordance with in vivo data, that bioavailability depends on (i) the nature and the physicochemical form of the actinide and (ii) the nature of the ligands in the static or dynamic phases [34,35]. In addition, the data obtained from this assay with An was found to show good correlation between dissolution data and urinary excretion following contamination in rat [34,36].

The present study aims at evaluating protein-An interactions in different physiologically relevant environments. For this purpose, we used the previously developed in vitro assay which provides more complex environments than most currently used in vitro methods, i.e., the presence of inorganic anions (carbonate and phosphate), physiologic pH, and temperature. This system may reveal the order of magnitude of affinity constants between exogenous ligands and the actinides, as well as more complex phenomena linked to An speciation. The present study will focus on two actinides (Pu and Am) and two iron-binding proteins (Tf representing a carrier protein in the blood, and ferritin, involved in the intracellular retention of iron in the liver). Notably, ferritin and transferrin differ significantly for their mode of interaction with iron. Transferrin binds Fe(III) synergistically with carbonate at two mononuclear binding sites, located in its two lobes [37]. Ferritin is a self-assembled 24-mer of two subunits (L and H) that interacts with Fe(II), oxidizes it into Fe(III) at ferroxidase sites (located in the H subunits), and can store thousands of Fe(III) ions in the protein as well as in an internal ferrihydrite mineral core [38,39]. In addition, we evaluated the ability of DTPA, the recommended chelating drug for treatment of internal contamination with Pu and Am, to compete with these proteins and to enhance An transfer in the various experimental conditions.

## 2. Materials and Methods

### 2.1. Reagents

NaCl and KCl were obtained from Sigma-Aldrich (France), Dulbecco‘s Phosphate buffered saline (DPBS) without Ca/Mg from Thermo Fisher scientific. Apo-Transferrin (76–81 kDa, T1147) and Ferritin from equine spleen (440 kDa, F4503) were from Sigma-Aldrich (France).

Marketed Ca-Diethylene Triamine Pentaacetic Acid (DTPA) solution as the calcium trisodium salt (Na_3_[Ca-DTPA]) was provided by Pharmacie Centrale des Armées (PCA, France).

### 2.2. Actinide Compounds

Both Pu (12.5% ^239^Pu and 86% ^238^Pu in activity, and 97.5% ^239^Pu and 2.4% ^238^Pu in mass) and Am (99.8% ^241^Am and 0.2% ^238^Pu in activity, and 99.9% ^241^Am and 0.1% ^238^Pu in mass) were from CEA, France. Pu and Am nitrate were obtained after evaporation of a stock solution in 2N HNO_3_. For unbound compounds, Pu and Am were resuspended in water. When protein-actinide complexes were introduced in gels, evaporated actinides were resuspended in DPBS containing proteins so final concentration was 50 µM for transferrin and 10 µM for ferritin, unless otherwise indicated.

Each gel contained approximately 200 Bq corresponding to 11.3 ng (47 pmoles) for Pu and 1.9 ng (8 pmoles) for Am.

For the DTPA and Apo-Tf dose-response study, the Pu used contained about 94.8% of ^239^Pu and around 5.2% of the total alpha activity consisted of ^241^Am. However, this represented 99.9% of ^239^Pu in mass. Two hundred Bq (total alpha activity) corresponded to 1.14 µM ^239^Pu and 1.14 nM ^241^Am.

### 2.3. Preparation of Agarose Gels and Incubation Media

Agarose gels (2.5 % w/w) were prepared by dissolution of agarose powder type IIA (Sigma, France) in the physiological saline NaCl/KCl (140 mM NaCl, 5 mM KCl, pH 5.6) or in phosphate buffered saline 137 mM NaCl, 2.7 mM KCl, 1.5 mM KH_2_PO_4_, 8.1 mM Na_2_HPO_4_ (DPBS). In addition to bone, phosphate is found in the blood (around 1 mM), and intracellular concentrations range from 0.5 to 5 mM [40]. The agarose solution was melted by heating in a microwave. After 5 min cooling at room temperature, the actinide to be tested was added under gentle stirring. The agarose solution was then distributed in 24 well-culture dish (300 µL/well). After the gel had solidified, which represents the static phase, the dynamic phase (buffer +/− ligands) was added to the wells (700 µL/well). For all experimental conditions, the plates were then incubated at 37 °C in a 5% CO_2_ atmosphere, to provide bicarbonate anions, and on an orbital shaker to prevent settling of the radio-contaminant. Under a PCO_2_ = 5% atmosphere, considering that the phosphate buffer maintains the pH at 7, and taking into account the Henri law ([CO_2_(aq)] = KCO_2_PCO_2_ with KCO_2_ = 3 × 10^−2^) for the dissolution of CO_2_ into CO_2_(aq) and the equilibrium constant K_A_ = [H^+^][HCO_3_^−^]/[CO_2_(aq)] = 8 × 10^−7^ for a culture medium at 37 °C, we can estimate a concentration of bicarbonate HCO_3_^−^ of around 12 mM in the in vitro assay with DPBS or the NaCl/KCl solution.

All conditions were done in triplicate. Dynamic phases were of the same composition as the gels and contained or not additional molecules (ApoTf, ferritin, DTPA).

In each individual experiment, controls were represented by the contaminant added to gels prepared in saline or DPBS and incubated in dynamic phases of same composition without additional ligand.

### 2.4. Collection of Fluids and Activity Measurement

After two hours of incubation, the dynamic phase was collected and placed in a scintillation counting glass vial. A measure of 700 µL of fresh dynamic phase of the same composition was added to replace the collected samples. The plates were further incubated for 22 h, after which similar collection process occurred. Then, 48 hours after the beginning of the incubation, the dynamic phase was collected as described above. The gels were removed from the wells and placed in glass vials. Gels were dried and wet-ashed with nitric acid. Activity (in Bq) was then measured by liquid scintillation counting (TriCarb 2500, Perkin Elmer, Villebon-sur-Yvette, France) in the presence of Ultima Gold^TMAB^ (Perkin Elmer, France).

### 2.5. Data Processing

Total recovered activity was calculated as the addition of the activity measured in each well (sum of activity from the dynamic phase fluid at the 3 time points and the activity found in the gel at the end of experiment). Each data point represents cumulative fraction of transferred activity measured in the dynamic phase at each sampling time/recovered activity deposited in each well. In all experimental conditions, the recovered activity represents more than 95% of the initial activity deposited in gels, so will be referred to as initial activity thereafter.

Box-and-whisker plot representation was used to illustrate the distribution of the dataset [41]. Box plots display the full range of variation (from minimum to maximum), the likely range of variation (the IQR), and a typical value (the median). The upper limit of the box represents the upper quartile, and the lower limit, the lower quartile. The error bars represent the upper and lower extremes. The horizontal bar inside the box represents the median value. The dots represent outliers, either 3×IQR or more above the third quartile or 3×IQR or more below the first quartile. This graph representation was chosen as it provides a graphical display of the distribution of results as well as indications of symmetry within the data.

For datasets of smaller size, results were expressed as mean ± standard deviation (SD).

Enhancement factor calculation: the enhancement factor of Pu or Am transfer obtained in each treated well was calculated as the ratio of medians (treated/untreated).

Statistical analysis was performed using Sigma Plot 14.5 (Ritme). Individual differences were assessed with non-parametric Mann–Whitney tests. Differences were considered significant at *p* < 0.05.

## 3. Results

### 3.1. Influence of Phosphate in the Static Phase on Pu and Am Transfer

It is widely admitted that actinides can form complexes with various inorganic and organic ligands (for review, see [1]). Using our biphasic assay, we first evaluated the influence of an inorganic ligand on the ability of Pu and Am to be transferred from a static phase (agarose gel) to the fluid phase. Phosphate was used to mimic in part blood or interstitial fluid.

With this assay, we previously showed that transfer from static to dynamic phase reflects the bioavailability of the actinide and its in vivo behavior [34]. For this purpose, gels were prepared in NaCl/KCl or in phosphate buffer, DPBS, as described in the materials and method section. Actinides were added to the gel, before gel polymerization, and the gels were then incubated at 37 °C in a 5% CO_2_ atmosphere in a solution identical to the one used for agarose gel preparation. The extent of An transferred to the liquid phase was measured at H2, H24, and H48.

**Figure 1:**
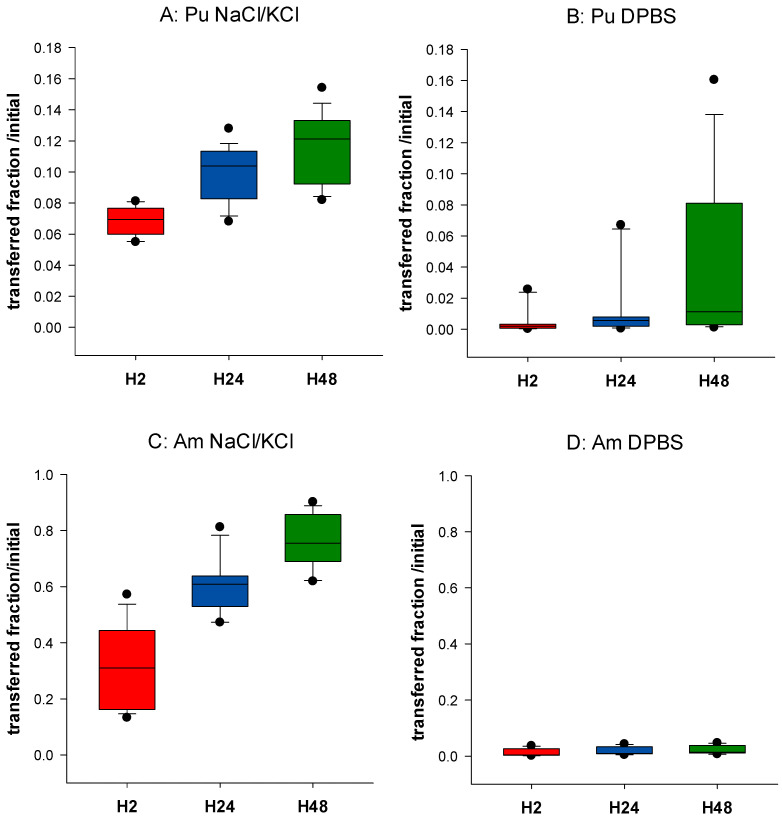
Influence of phosphate on Pu and Am transfer. Pu (**A**,**B**) or Am (**C**,**D**) were included in the static retention phase (agarose gels) prepared in NaCl/KCl (**A**,**C**) or in DPBS (**B**,**D**). After 2, 24, and 48 h incubation in NaCl/KCl or DPBS, the transferred activity was measured in the dynamic phase using liquid scintillation counting. Results are expressed as cumulative activity/initial activity in the gel. Each experimental condition was done in triplicate and repeated 6 times.

Figure 1 shows the behavior of Pu and Am in NaCl/KCl or DPBS. Differences between Pu and Am are observed. Up to 81% (median value) Am is transferred from the gel in the physiological saline solution after 48 h of incubation (H48), while only 12% of Pu is transferred in the same conditions (Figure 1A,C). These data are in accordance with previous experiments [34]. The retention of Pu and Am in the static phase prepared in phosphate buffer was much larger than that prepared in simple saline solution, with not more than 1.1% of Pu and 2.2% Am released after 48 h (Figure 1B,D). A decrease in transfer to the dynamic phase by 36-fold for Pu and 78-fold for Am is observed at H2, and by 11-fold for Pu and 55-fold for Am at H48. These results indicate the possible formation of complexes between An and phosphate within the agarose gels, which limits their transfer. This is in accordance with the well-known very strong chemical affinity of actinides for phosphate groups, whether of inorganic or organic origin. The difference in transfer in the presence or not of phosphate is smaller for Pu than for Am. However, this can be correlated with an already smaller transfer rate of Pu in the physiologic saline as compared to Am.

### 3.2. Influence of Various Ligands Added in the Dynamic Phase on the Transfer of Pu/Am

#### 3.2.1. Transfer of Pu/Am in the Presence of a Synthetic Ligand (DTPA) According to the Composition of the Static Phase

In our previous work, we demonstrated that transfer of Pu and Am from the static phase prepared in saline solution is increased in the presence of exogenous ligands, such as DTPA added in the dynamic phase [34,35]. This suggested that a diffusion of DTPA from the dynamic to the static phase occurred, allowing the formation of actinide-DTPA complexes within the gels, followed by a rapid transfer to the dynamic phase and elimination during the replacement of the dynamic phase at each collection time. The replacement of the fluid phase will consequently favor more transfer of An in the dynamic phase, until the An is totally transferred.

**Figure 2:**
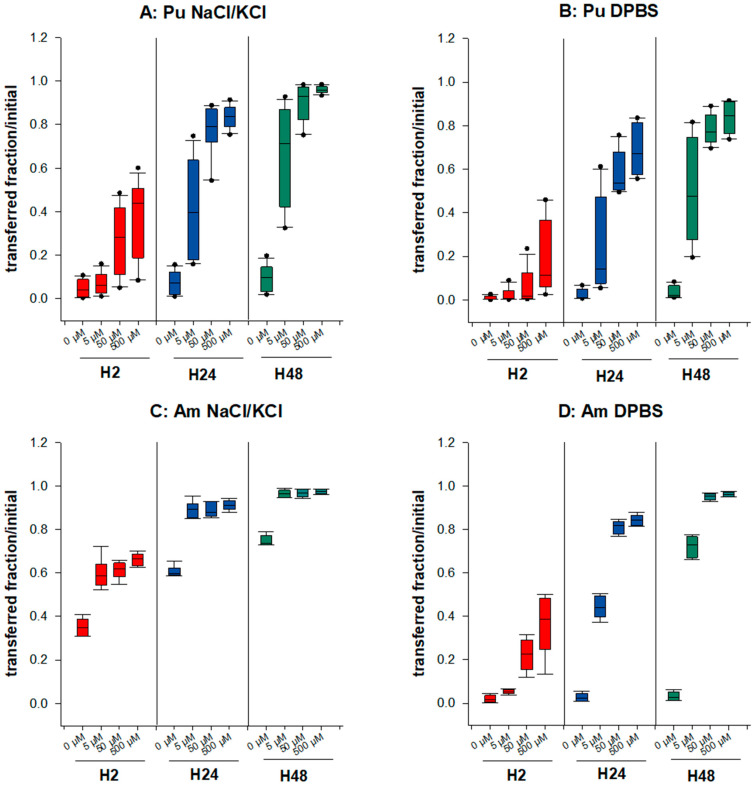
Transfer of Pu/Am in the presence of a synthetic ligand, DTPA. Pu (**A**,**B**) or Am (**C**,**D**) were included in the static retention phase (agarose gels) prepared in NaCl/KCl (**A**,**C**) or in DPBS (**B**,**D**). After 2, 24, and 48 h incubation in NaCl/KCl or DPBS supplemented or not with DTPA (5, 50, 500 µM), the transferred activity was measured in the dynamic phase using liquid scintillation counting. Results are expressed as cumulative activity/initial activity in the gel. Each experimental condition was done in triplicate and repeated 4 times for Pu in NaCl/KCl, 3 times for Pu in DPBS, and twice for Am in NaCl/KCl or DPBS.

The underlying question here is whether the speciation of Pu/Am in a retention compartment influences the bioavailability to chelating agents. Thus, we compared the transfer of Pu and Am from a static phase made with NaCl/KCl or DPBS to the dynamic phase where DTPA is added at various concentrations (5–500 µM). A time-related dose-response relationship is observed whatever the experimental condition tested (Figure 2). The mobilizing effect of DTPA on Pu transfer in the saline solution is very similar to our previous results [34]. The transfer to the mobile phase is increased by a factor up to 10 for the highest DTPA concentration at H48 for Pu, and 1.3 for Am. For Am, the addition of 5 µM DTPA is already sufficient to obtain nearly maximal mobilization (>95% transferred at H48), while for Pu, at least 50 µM DTPA is necessary to reach a similar effect. In DPBS, the mobilizing effect of DTPA for Am is higher than in NaCl/KCl. This could be explained, at least partly, by the greater mobility of the An in the NaCl/KCl medium, with almost the totality of Am being transferred at H48 in the presence of 500 µM DTPA, which is not the case in phosphate-containing static phase. As a consequence, the important difference in the bioavailability of both Pu and Am between static phases made with or without phosphate observed in the absence of DTPA becomes modest in the presence of DTPA.

Differences observed between Pu and Am are illustrated in Table 1. In NaCl gels, the increased transfer in the presence of DTPA is higher for Pu than for Am. As an example, 4.5 times more Pu is transferred in 2 h in the presence of 50 µM DTPA than in the absence of DTPA whereas a 1.8 increase is observed in the same condition for Am (Table 1), possibly due to the higher spontaneous transfer of Am in the dynamic phase without DTPA. In contrast, in gels containing phosphate, the DTPA-induced increase in transfer is higher for Am than for Pu (5.5-fold increase for Pu and 13.2 for Am at H2). Differences between Pu and Am are still observed at the later time points. These findings suggest that the speciation of actinides in a retention compartment has major consequences on their availability to chelating agents.

#### 3.2.2. The Presence of Bioligand (Tf) in the Dynamic Phase Influences the Transfer of Pu in the Absence of Phosphate

**Figure 3:**
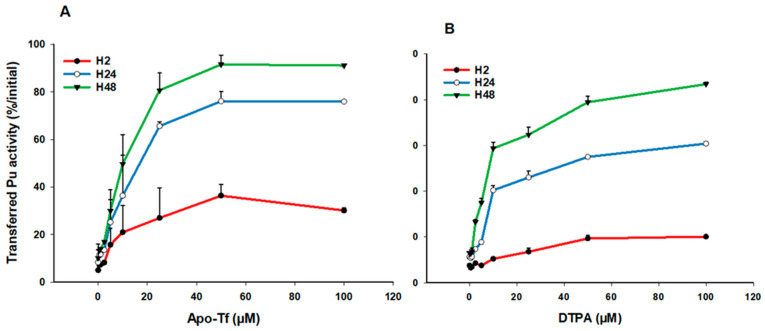
Transfer of Pu in simple saline solution in the presence of ApoTf or DTPA. Pu was included in the static retention phase (agarose gels) prepared in NaCl/KCl. After 2, 24, and 48 h incubation in NaCl/KCl supplemented with increasing concentrations of ApoTf (**A**) or DTPA (**B**), the transferred activity was measured in the dynamic phase using liquid scintillation counting. Results are expressed as cumulative activity/initial activity in the gel at the various times, according to ApoTf or DTPA concentrations. Each experimental condition was done in triplicate and experiments were repeated once for DTPA and twice for ApoTf.

Next, we evaluated the ability of the main blood carrier protein, transferrin (Tf), to increase the transfer of Pu in the dynamic phase. We used a wide range of ApoTf concentrations (0.5–100 µM) reflecting physiological concentrations in human fluids [42]. Figure 3 shows the ApoTf (3A) or DTPA (3B) dose-response relationship at the various time points for gels made in physiological saline. ApoTf significantly increases the transfer rate of Pu in the liquid phase, up to 30% within 2 h and up to 80–85% at H48. Interestingly, whatever the incubation time, a plateau seems to be reached for Pu transfer, with added ApoTf concentrations between 25 and 50 µM, which corresponds to the concentration found in serum. A plateau at around 50 µM is also observed for DTPA. This behavior strongly suggests an ability of ApoTf to diffuse into the gel and chelate Pu, similarly to DTPA.

#### 3.2.3. Influence of Bioligands (ApoTf, ferritin) or Synthetic Ligands (DTPA) on Pu/Am Transfer in the Presence of Phosphate

To better characterize the ability of Pu and Am to form complexes with proteins in controlled and physiologically relevant media, we added ApoTf or ferritin in the dynamic phase and we evaluated their capacity to enhance transfer of Pu/Am from a static phase made with DPBS. ApoTf concentration used was near the concentration in human serum (≈30 µM, [42]) and that of ferritin was of 10 µM, considerably higher than in the serum which is approximatively around 20 nM) [43].

**Figure 4:**
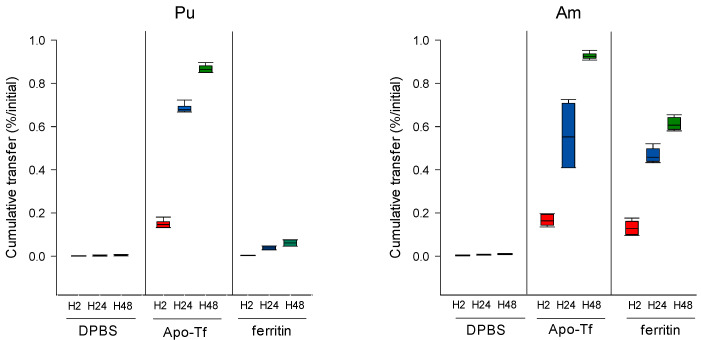
Pu/Am transfer in the presence of proteins in the dynamic phase. Pu or Am were included in the static phase prepared in DPBS. After 2, 24, and 48 h incubation in DPBS supplemented with ApoTf (50 µM) or ferritin (10 µM), the transferred activity was measured in the dynamic phase using liquid scintillation counting. Results are expressed as cumulative activity/initial activity in the gel. Each experimental condition was done in triplicate and repeated twice.

Our results indicate that in the presence of ApoTf or ferritin in the dynamic phase, an increased transfer is observed for both Pu and Am (Figure 4) as compared to transfer in the absence of proteins. However, the importance of the effect varies with both the actinide and the ligand. Table 2 reports the enhancement factors calculated in the different experimental conditions. Data obtained with DTPA (50 µM) shown in Figure 2 are also included in Table 2 for comparison. ApoTf increases Pu transfer by 437-fold at H48 as compared to DPBS alone, and Am by 98-fold (Table 2). Pu and Am transfer in the presence of ApoTf reflects approximately 90% of the initial activity. In contrast, in the presence of ferritin, only 6.14% of Pu is transferred as compared to 60.7% for Am. These results are in line with the higher affinity that has been reported for Tf towards Pu(IV) as compared to Am(III) [1,24,25]. They also suggest an opposite situation for ferritin, with a stronger affinity for Am(III) than for Pu(IV) in the DPBS experimental conditions.

At H2, ApoTf is much more effective in increasing Pu transfer than DTPA at the same concentration of 50 µM (with enhancement factors of 308.3 vs. 16.5, Table 2). For ferritin, although direct comparison is not possible since the ferritin concentration is 10 µM only, it seems that the protein is less efficient in chelating Pu than ApoTf. For Am, ApoTf was slightly less efficient than DTPA to enhance the transfer in the liquid phase. Given the lower concentration of ferritin added to the assay, ferritin could be more efficient in Am transfer than ApoTf, and at least as efficient as DTPA. At this early time point, the ApoTf effect on Pu transfer is higher than on Am, whereas ferritin and DTPA have a higher influence on Am than on Pu. At later time points, we observed similar trends for Am, as compared to H2. However, for Pu the enhancement factor increases with time and the most prominent time-related effect is observed in the presence of DTPA (over 20 times higher at H48 as compared to H2).

### 3.3. Behavior of Actinide/Protein Complexes: Transfer from Static to Dynamic Phase

#### 3.3.1. In the Absence of Bioligands in the Dynamic Phase

In the previous sections, we showed that the addition of ligands in the dynamic phase of our assay increased the transfer of actinides. Next, we studied the transfer of preformed complexes contained in the gels. For this purpose, preformed An/protein complexes were added in gels made with DPBS, and the transfer of these complexes were compared to that of unbound Pu/Am at the different timepoints. A slight but not statistically significant increase in transferred activity is observed for Pu-ApoTf and Pu-ferritin preformed complexes as compared to unbound Pu (*p* < 0.05, rank sum test, Table 3). The enhancement factors increase slightly for Pu complexes at later time points but remain non-significant. The moderate transfer enhancement of actinide-protein complexes as compared to unbound actinides suggests that actinide-protein complexes retain the ability to bind to phosphate ions.

Similarly to Pu-ApoTf, Am-ApoTf complex transfer is not significantly augmented as compared to unbound Am. A slight decrease in the enhancement factor is even observed over time (Table 3). In contrast, the activity from Am-ferritin complex is transferred 26 times more than for the unbound Am. At the later time point, only small changes are observed, with over 20 times more activity transferred for the Am-ferritin complexes. The higher molecular weight of ferritin is 440 kDa, compared to 80 kDa for Tf, which could suggest a slower mobility of the Am-ferritin complex as compared to the Am-ApoTf within the gel. The lower mobility of ferritin could also be related to the large size of the protein structure (12 nm [44]).

However, it is presently too early to identify under which form Am is transferred from the static to the dynamic phase, and whether the Am-ferritin complex dissociates within the gel, promoting Am transfer, or whether the complex moves out of the gel.

#### 3.3.2. In the Presence of Ligands in the Dynamic Phase

To elucidate whether the addition of bio- or synthetic ligands in the dynamic phase would facilitate the transfer of the An for an An-protein complex in the static phase to a soluble form in the dynamic phase, we added either proteins or DTPA in the dynamic phase. Results from Table 4 indicate that Pu from the Pu-ApoTf complexes can be further mobilized from the static phase by adding ApoTf or DTPA in the dynamic phase and to a greater extent with ApoTf. Similarly, Am transfer is increased in the presence of ApoTf or DTPA in the dynamic phase, and as previously observed (Table 2), the effect of DTPA is larger than that of ApoTf. Concerning Pu or Am released from the An-ferritin complexes included in the static phase, the presence of ligands in the dynamic phase favors their transfer with a lower magnitude for ferritin 10 µM than for DTPA 50 µM.

**Figure 5:**
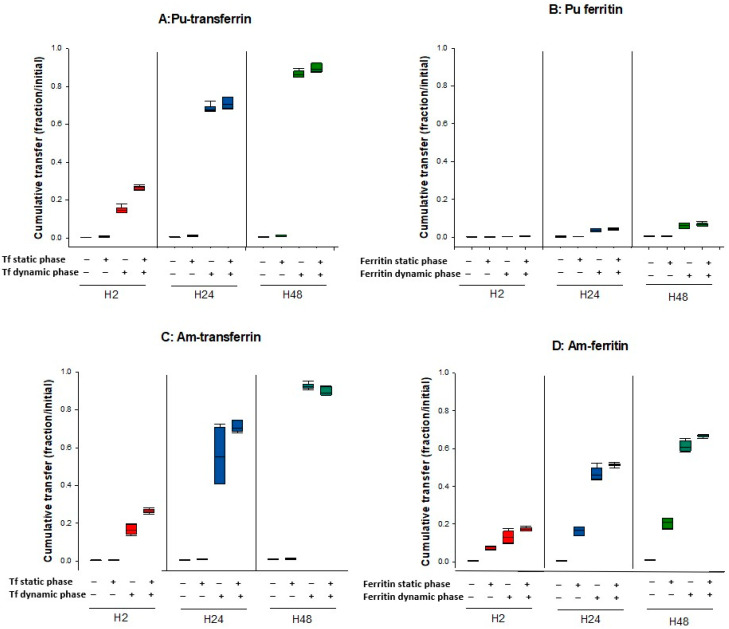
Transfer of Pu/Am in the presence or not of ligands in the static or dynamic phase. Pu (**A**,**B**) or Am (**C**,**D**) were resuspended in water or in ApoTf or ferritin and included in the static retention phase (agarose gels) prepared in DPBS. ApoTf 50 µM or ferritin 10 µM were added in the dynamic phase or not. The – and + signs indicate the absence or the presence of protein in a specific phase. After 2, 24, and 48 h incubation in DPBS, the transferred activity was measured in the dynamic phase using liquid scintillation counting. Results are expressed as cumulative activity/initial activity in the gel. Each experimental condition was done in triplicate and repeated twice.

Figure 5 reports the differential influence of ApoTf and ferritin on Pu and Am transfer in various experimental conditions, and notably by changing the compartment in which the bioligand is present. The results shown are 1—the transfer of Pu and Am in the absence of exogenous ligands in the dynamic phase, 2—the transfer of Pu and Am when proteins are added in the dynamic phase, and 3—the transfer of Pu and Am with An-protein complexes in the static phase in the presence or not of proteins in the dynamic phase.

Adding an exogenous ligand favors the transfer of Pu and Am whatever their initial state in the static phase (complexed with proteins or “unbound”). However, the extent to which this phenomenon occurs depends on which ligand is present and where (Figure 5).

The total transfer efficiency of Pu from the gel to the dynamic phase using DTPA, Apo-Tf, or ferritin is very similar whatever the form of Pu deposited in the gel, i.e., deposited alone (Table 2) or in the form of a Tf-Pu or ferritin-Pu complex (Table 4). For example, 86.3 % (Table 2) or 88.8% (Table 4) Pu was transferred with ApoTf at 50 µM at H48, and 6.1% (Table 2) or 6.9 % (Table 4) Pu was extracted at H48 with ferritin at 10 µM. For Am, the behavior is different. The same percentage of Am is released in the dynamic phase with DTPA, whatever the form of Am in the gel with values ranging from 47.4 to 50.2% at H2, 81.5 to 83.7% at H24 and 93.9 to 94.6% at H48 (Table 2 and Table 4).

Results indicate that Pu and Am have similar behavior towards ApoTf. These behaviors include: (i) the low mobilization of actinide-Tf complexes in the absence of ApoTf in the dynamic phase and (ii) the high mobilizing effect of ApoTf towards Pu and Am whether complexed or not to ApoTf. The increased transfer is observed more rapidly for the actinide-ApoTf complexes than for the unbound actinides, but the overall transfer after 48 h is quite similar in both cases.

Finally, concerning ferritin, a very different behavior towards Pu and Am can be observed. The influence of ferritin on Pu transfer is modest whatever the experimental conditions, as compared to the effect of ApoTf, even considering the difference in the concentrations used. In contrast, ferritin seems to display a high affinity towards Am, which favors its transfer out of the gel in the absence of ligands in the dynamic phase. It also facilitates transfer of Am when ferritin is present in the dynamic phase, with an increased Am transfer (up to 67% at H48).

## 4. Discussion

The present study was undertaken to better characterize interactions of Pu and Am with various ligands in biologically relevant environments and to evaluate whether the differential ability of Pu and Am to bind to bioligands (phosphate and metal-binding proteins) could, at least partly, explain their different biological behavior. To this end, we used a previously developed acellular in vitro assay which importantly can reflect the in vivo behavior as observed after pulmonary administration in rats [36]. This dynamic assay can be described as a multi-compartmental model, mimicking the multiple retention and transfer compartments in the body. Whereas other elegant techniques such as Atomic Force Microscopy [45,46] have been used to study metal-protein interactions in physiologically relevant environments, none of these methods have been applied to the strongly radioactive alpha particle emitters such as Pu and Am. Following in vivo uptake, biodistribution of actinides reflects a multi-stage process, integrating the dissolution of the contaminant, the interactions with biological ligands, cellular uptake (macrophages), and transfer into the circulation (blood, lymph, interstitial fluid). Although Pu and Am both accumulate mainly in liver and bone after their blood transfer, some differences have been reported between the two elements, such as an accelerated transfer from lungs to blood for Am and a shorter retention time in the blood [2]. The different behaviors of Pu and Am in vivo and in vitro, might not be only due to differences in their ionic radius, but also and importantly to their oxidation state and to the different stability of the complexes that they form with various (bio)ligands. While Am is expected to be present mainly in the Am(III) state, Pu has a complicated speciation and can coexist in several oxidation states in solution (Pu(III), Pu(IV), or Pu(V) as PuO_2_^+^), depending on pH, presence of ligands, etc. However, Pu(IV) is mostly expected in vivo. In addition, as Fe(III), Pu(IV) has a strong tendency to hydrolyze and to form insoluble hydroxides [47]. These differences in redox state and chemical properties result in differences in interactions of Pu and Am with bioligands and may also explain the difference in DTPA decorporation efficacy observed in vivo.

Similar to ICRP biokinetic models describing the behavior of radionuclides in the body, our assay mimics transfer from a retention compartment to another one. The form under which An is retained in a compartment (cells, extracellular fluids, tissue, etc.) may vary with the composition of the considered compartment. Thus, a complex formed in one compartment may encounter other potential ligands in another compartment, in which competition between ligands may occur. This situation can be further complicated when exogenous ligands are added such as chelating agents used for actinide decorporation (DTPA). Using our biphasic assay, we can partly reproduce these complex situations. The results reported here demonstrate the differential influence of the composition of both static and dynamic phases on the behavior of Pu and Am.

We first evidenced the influence of phosphate buffer on the bioavailability of Pu and Am, i.e., their ability to be transferred from the static to the dynamic phase. In the presence or not of phosphate, Am bioavailability is higher than that of Pu, reflecting higher dissolution properties for Am [2]. Despite the presence of carbonate in the assay, in the absence of strong chelating ligand, Pu(IV) could form insoluble hydroxide colloids in the gel. The presence of phosphate in the static phase markedly limits the transferability of both Pu(IV) and Am(III) but to a higher extent for Am. This is in line with previous data showing the higher retention of Am by the calcium phosphate mineral, hydroxyapatite compartment of bone, as compared to Pu [35,48]. Secondly, we showed that the presence of phosphate in the static phase limits the efficacy of DTPA on Pu transfer whereas an opposite situation is observed for Am. Phosphate and DTPA are in competition for the formation of Am complexes and higher amounts of DTPA are needed to transfer the quasi totality of Am in the dynamic phase. More importantly, our data show different DTPA efficacy depending on the speciation of An.

In addition to inorganic anions, proteins are important biomolecules involved in the complexation of metals, including An, and represent potential binding sites for their sequestration in cells and tissues. Thus, proteins are expected to play important roles in determining the biological behavior of An. It is now admitted that, in the lungs, a bound fraction of Pu, possibly on proteins, is involved in a protracted release into the circulation with consequences on dose calculation [49,50]. Tf is the candidate protein for this behavior, as it is present in lung epithelial lining fluid [51].

The interactions between An and protein have also been identified as a key event in cellular uptake in various experimental models, either favoring [23] or inhibiting cellular uptake [15,52]. Hence, the precise mechanism involved in cellular uptake remains controversial. This is possibly due to the diversity of the experimental conditions and models, and more particularly of cell lines used for these studies. Our biphasic acellular assay may represent an interesting approach to overcome these discrepancies because we strictly control the composition of each compartment. In addition, although it is usually recognized that Pu(IV) forms much stronger complexes with proteins than Am(III), only rare studies have been dedicated to the thermodynamic comparison of Pu(IV) /Am(III) protein interactions. Such data are nonetheless important, considering the differences in the behavior of Pu and Am in vivo. Transportation of Pu and Am to various organs occurs mainly through metal-binding proteins, such as the iron-carrying proteins Tf or ferritin. Tf has been identified as the main carrier protein for Pu in the blood with 90% of Pu being associated to Tf in the serum [22]. This percentage drops to approximately 30% for Am, for which complexes of weaker affinity, such as serum albumin, have also been identified [53,54]. In addition to their role as carrier proteins in the blood, metal-binding proteins may also be involved in the long-term intracellular retention of actinides. This is particularly the case for ferritin, the protein responsible for intracellular iron storage and which is present in cytosol of various cell types, such as liver cells and macrophages. Ferritin has been shown to be associated with Pu and Am retention in the lysosomes of hepatic cells [20,55]. Although no such demonstration has been made in macrophages to our knowledge, ferritin might also be involved in actinide retention in these phagocytic cells. Long-term damage might result from An retention, such as inflammatory reaction.

We thus focused our study on Tf and ferritin, to be representative of two important compartments in the body after An intake: blood and intracellular milieu. While some data exist for Tf interactions with Pu(IV) and Am(III), there are no data on the stability constants of ferritin with Pu(IV) and Am(III) nor clear information on the number of An ions that can be stored by this oligomeric Fe(III) storage protein.

We first showed that both proteins favor the transfer of Pu and Am from the static to the dynamic phase, thus confirming the strong affinity of Tf and ferritin towards Pu and Am. However, a dual differential effect was observed: 1—for one protein and both An (Ferritin) and 2—for two proteins and one An (Am). Hence, the transfer enhancement of Pu in the presence of ApoTf is larger than for Am, which is in agreement with older studies where Am-Tf complex appears to be less stable than those formed with Pu(IV) [53,54]. In contrast, the ferritin-induced transfer is higher for Am(III) than for Pu(IV). When comparing the effect of the two proteins on Pu transfer, we show that transferrin has a larger effect than ferritin. This differs from previously published results suggesting that Pu forms a more stable complex with ferritin than with Tf [21]. However, recent data may be in line with these results. Pu(IV) was shown to interact at the Fe(III) binding sites of Tf, with high stability (logK = 24–26, [24,25]). As mentioned above, there are no thermodynamic data concerning the Pu(IV)-ferritin interaction, and such thermodynamic analysis may be complicated since, given the ferritin:iron stoichiometry, a large number of Pu(IV) atoms may interact with ferritin. However, recent theoretical and XAS structural studies of ferritin-Pu(IV) interactions concluded that Pu(IV) binding sites may be rather exposed, at conserved Asp and Glu residues of the L subunit, with carbonate and/or water ligands completing the Pu(IV) coordination sphere. In addition, data from EXAFS analyses found no trace of Pu(IV) associated with the ferrihydric core of ferritin [29,56]. These data suggest that the Pu-ferritin complexation may largely depend on Pu(IV) speciation. The similar efficacy of ApoTf and ferritin in enhancing Am transfer, suggests a higher stability of the ferritin-Am(III) complex as compared to Pu(IV) and that Pu(IV) and Am(III) bind to ferritin by different mechanisms. This behavior may be due to the oxidation state of Am(III) versus Pu(IV) or to its different speciation in solution.

The biphasic condition of our assay allows the addition of proteins in either the static or dynamic phase, thus mimicking situations where the proteins are present in a retention compartment (retention organ such as lungs or liver) or in a transfer compartment (blood). It was thus possible to evaluate the importance of the compartment in which the ligand is present. For Pu-ApoTf, Pu-ferritin, and Am-ApoTf preformed complexes added in the static phase, no effect on transfer is observed, which contrasts to the transfer observed for Pu or Am nitrate in the absence of exogenous ligands in the dynamic phase. This may be due to a diffusion-limited mechanisms, since the proteins are large macromolecules of 80 kDa for transferrin and 440 kDa for ferritin. However, a different behavior is observed when preformed Am-ferritin complexes are added in the static phase. In this case, transferred activity is more than 20 times higher than that of Am nitrate. Although the approach used does not allow the determination of which form of Am is transferred (complexed or not), we can hypothesize that a dissociation of the Am-ferritin occurs within the agarose gel, freeing Am and allowing the transfer into the dynamic phase. However, this hypothesis does not corroborate the irreversible property of Am-ferritin complex described by Stover et al. [20]. Nonetheless, in a recent study in rats, intracellular chelation with DTPA in liver has been shown to be more important for Am than for Pu suggesting that, since ferritin is present in lysosomes of liver cells, DTPA dissociates Am-ferritin complexes more readily than Pu ferritin complexes [57]. Given our results of the Pu-ferritin complex, a different speciation of Pu(IV) may also be present in cells. Nevertheless, of particular interest is the specific behavior of Am(III) towards ferritin as compared to Pu(IV). Further investigations are needed to explore the underlying mechanisms.

Finally, we evaluated the ability of DTPA to compete with protein-An complexes. For this purpose, DTPA was added in the dynamic phase over gels containing preformed An-protein complexes. Interestingly, whatever the experimental conditions (presence of phosphate, protein-actinide complexes), DTPA enhances transfer from static to dynamic phase. For both Pu-Tf and Pu-ferritin complexes, DTPA increases transfer with a similar efficacy (approximately 75-fold increase, Table 4). This may reflect the capacity of DTPA to dissociate Pu(IV) from already-formed complexes. In the case of proteins added in the dynamic phase, additional complexes could be formed without the need of dissociating existing complexes. In favor of this hypothesis is the slower time-course of DTPA-induced effect as compared to the Tf- or ferritin-induced transfer. Transfer of Am-Tf in the presence of DTPA follows the same pattern confirming an older study demonstrating that DTPA can displace Pu and Am from complexes with Tf [58].

## 5. Conclusions

The main objective of the present study was to evaluate An interactions with various ligands in complex physiologically relevant environments. Our results indicate that a recently developed biphasic and dynamic assay represents a useful tool to explore interactions between actinides and ligands of various origins (synthetic, inorganic, organic). By modifying the composition of the static and dynamic phases, we demonstrated the possibility of using our assay to study competition between various ligands. We also evidenced the role of the An speciation as well as differences in the formation of actinide-protein or actinide-DTPA complexes, demonstrating the specific behavior of Pu(IV) and Am(III). Of great interest is the specific behavior of the Am(III)-ferritin interactions.

We believe that our assay can at least partly reproduce the in vivo behavior of An and could be used to investigate in relevant physicochemical conditions, An interactions with proteins recently described as potential as ligands as well as to evaluate the efficacy of novel drug candidates for decorporation.

## Figures and Tables

**Table 1:** Pu/Am transfer enhancement in the presence of DTPA. Ratio of medians of transferred fractions from DTPA treated vs. untreated obtained from 2–4 experiments in triplicate.

Dynamic Phase	Static Phase Pu						Static Phase Am					
	NaCl			DPBS			NaCl			DPBS		
	H2	H24	H48	H2	H24	H48	H2	H24	H48	H2	H24	H48
NaCl/KCl DTPA 50 µM	4.5	54.5	37.1	-	-	-	1.8	1.5	1.3	-	-	-
DPBS DTPA 50 µM	-	-	-	5.5	11.0	11.6	-	-	-	13.2	34.2	34.9

**Table 2:** Pu/Am transfer enhancement in the presence of proteins or DTPA in the dynamic phase. Ratio of medians of transferred activity in dynamic phase supplemented with ligands vs. control (DPBS alone) and median of cumulative transfer (%). Results are from two experiments performed in triplicate.

Dynamic Phase		Static Phase Pu			Static Phase Am		
		H2	H24	H48	H2	H24	H48
DPBS + ApoTf (50 µM)	Fold increase/no ligand	308.3	518.4	437.2	56.0	80.4	98.3
	Cumulative transfer (%)	14.6	67.8	86.3	16.3	55.2	92.4
DPBS + ferritin (10 µM)	Fold increase/no ligand	7.1	28.7	31.1	43.8	66.8	64.5
	Cumulative transfer (%)	0.3	3.7	6.1	12.8	45.8	60.7
DPBS + DTPA (50 µM)	Fold increase/no ligand	16.5	314.8	357.8	171.9	121.9	100.7
	Cumulative transfer (%)	0.8	41.1	70.6	50.2	83.7	94.6

**Table 3:** Transfer enhancement of Pu/Am-protein complexes in the absence of ligands in the dynamic phase. Enhancement factor of transferred activity for protein-actinide complexes vs. uncomplexed “free” actinide (H2). Ratio of medians are from two experiments performed in triplicate.

Dynamic Phase	Static Phase Pu						Static Phase Am					
	Pu-ApoTf			Pu-Ferritin			Am-ApoTf			Am-Ferritin		
DPBS	H2	H24	H48	H2	H24	H48	H2	H24	H48	H2	H24	H48
	1.68	2.85	2.49	1.28	2.07	2.22	1.61	1.38	1.34	26.3	24.6	22.4

**Table 4:** Transfer enhancement of Pu/Am-protein complexes in the presence of ligands in the dynamic phase. Ratio of medians of transferred activity and cumulative transfer in the dynamic phase (%/initial activity) for protein-actinide complexes in the presence of ligands in the dynamic phase vs. in the absence of ligand. Values are from two experiments performed in triplicate.

Dynamic Phase		Static Phase Pu						Static Phase Am					
		Pu-Tf			Pu-Ferritin			Am-Tf			Am-Ferritin		
		H2	H24	H48	H2	H24	H48	H2	H24	H48	H2	H24	H48
DPBS + 50 µM ApoTf	Fold increase/ no ligand	215.4	188.1	180.9	-	-	-	55.5	70.1	67.1	-	-	-
	Cumulative transfer (%)	17.2	70.34	88.8	-	-	-	26.2	73.4	91.2	-	-	-
DPBS + 10 µM ferritin	Fold increase/ no ligand	-	-	-	6.1	12.9	13.5	-	-	-	2.3	3.0	3.2
	Cumulative transfer (%)	-	-	-	0.5	4.1	6.9	-	-	-	17.6	51.2	66.8
DPBS + 50 µM DTPA	Fold increase/ no ligand	38.9	132	150.8	30.2	148.2	142.7	100.6	77.8	69.1	6.5	4.9	4.5
	Cumulative transfer (%)	3.1	49.3	74.1	2.4	46.7	72.85	47.4	81.5	93.9	49.6	82.5	94.4

## Data Availability

Not applicable.
